# Epidemiology, clinical features and outcomes of hospitalized patients with COVID-19 by vaccination status: a multicenter historical cohort study

**DOI:** 10.1186/s12985-024-02325-x

**Published:** 2024-03-21

**Authors:** Shatha Alshanqeeti, Susan Szpunar, Premchand Anne, Louis Saravolatz, Ashish Bhargava

**Affiliations:** 1 Department of Internal Medicine, Ascension St. John Hospital, 19251 Mack Avenue, Suite 340, 48236 Detroit, MI USA; 2 Department of Biomedical Investigations and Research, Ascension St. John Hospital, Detroit, MI USA; 3 Department of Pediatrics, Ascension St. John Hospital, Detroit, MI USA; 4 Division of Infectious Disease, Department of Internal Medicine, Ascension St. John Hospital, Detroit, MI USA; 5 Thomas Mackey Center for Infectious Disease Research, Ascension St John Hospital, Detroit, MI USA

**Keywords:** COVID-19, Unvaccinated, Fully vaccinated, Risk factors, Severe disease, Breakthrough infections

## Abstract

**Introduction:**

COVID-19 disease resulted in over six million deaths worldwide. Although vaccines against SARS-CoV-2 demonstrated efficacy, breakthrough infections became increasingly common. There is still a lack of data regarding the severity and outcomes of COVID-19 among vaccinated compared to unvaccinated individuals.

**Methods:**

This was a historical cohort study of adult COVID-19 patients hospitalized in five Ascension hospitals in southeast Michigan. Electronic medical records were reviewed. Vaccine information was collected from the Michigan Care Improvement Registry. Data were analyzed using Student’s t-test, analysis of variance, the chi-squared test, the Mann-Whitney and Kruskal-Wallis tests, and multivariable logistic regression.

**Results:**

Of 341 patients, the mean age was 57.9 ± 18.3 years, 54.8% (187/341) were female, and 48.7% (166/341) were black/African American. Most patients were unvaccinated, 65.7%, 8.5%, and 25.8% receiving one dose or at least two doses, respectively. Unvaccinated patients were younger than fully vaccinated (p = 0.001) and were more likely to be black/African American (p = 0.002). Fully vaccinated patients were 5.3 times less likely to have severe/critical disease (WHO classification) than unvaccinated patients (p < 0.001) after controlling for age, BMI, race, home steroid use, and serum albumin levels on admission. The case fatality rate in fully vaccinated patients was 3.4% compared to 17.9% in unvaccinated patients (p = 0.003). Unvaccinated patients also had higher rates of complications.

**Conclusions:**

Patients who were unvaccinated or partially vaccinated had more in-hospital complications, severe disease, and death as compared to fully vaccinated patients. Factors associated with severe COVID-19 disease included advanced age, obesity, low serum albumin, and home steroid use.

## Introduction

As of August 16, 2023, Severe acute respiratory syndrome coronavirus-2 (SARS-CoV-2) led to 769,774,646 confirmed cases of COVID-19 and 6,955,141 deaths worldwide [1]. Since its declaration as a pandemic in March 2020, tremendous efforts have been put into developing therapies and vaccines to end the COVID-19 pandemic. In December 2020, a multinational, placebo-controlled, observer-blinded trial studied the efficacy and safety of the Pfizer-BioNTech vaccine in 43,548 participants. Among 170 cases of COVID-19 detected seven days after administering the second dose, eight patients were in the vaccine group, corresponding to 95% vaccine efficacy [2]. Another observer-blind, placebo-controlled trial conducted in the United States with 30,420 participants showed 94.1% efficacy of the mRNA-1273 vaccine [3]. In December 2020, two mRNA vaccines against SARS-CoV-2 from Moderna and Pfizer-BioNTech were authorized for emergency use by the Food and Drug Administration (FDA), followed by another viral vector vaccine from Johnson and Johnson in February 2021 [4]. On August 23, 2021, the two-dose Pfizer-BioNTech vaccine series (Comirnaty®) was approved by the FDA for the prevention of COVID-19 disease in individuals five years of age and older with a third primary dose for individuals who were immune compromised [5]. This was followed by the approval of the Moderna vaccine (Spikevax®) on January 31, 2022 [6]. Both approved vaccines were monovalent vaccines. Currently the bivalent Pfizer-BioNTech and Moderna vaccines are administered as primary vaccinations [5, 6]. Several studies demonstrated vaccine efficacy; however, the frequency of breakthrough COVID-19 infections started to increase. As of April 30, 2021, a total of 10,262 vaccine breakthrough infections were reported among the 101 million persons who were fully vaccinated persons at that time in the United States [7]. Another study conducted during January-April 2021 from Israel reported a rate of breakthrough infection of about 0.4% among symptomatic healthcare workers or those who had a known exposure to COVID-19 [8]. A retrospective study done in New York, USA, found the incidence of breakthrough infection to be 0.16 per 1,000 person-days. Males were more likely than females to have breakthrough infections. Chronic pulmonary disease, heart disease, immune deficiency, advanced age, anemia, and illicit drug use were among the risk factors associated with breakthrough infections [9–11]. Black race was associated with a lower risk of developing breakthrough COVID-19 infection [10]. Individuals who received the Moderna vaccine were at a lower risk of breakthrough infections than those who received the Pfizer-BioNTech vaccine in multiple studies. However, the effectiveness of both vaccines diminished over time [9–12].

In a systematic review of 969 patients who were hospitalized with COVID-19 infection, 797 were unvaccinated, 103 received only one dose of either the Moderna or Pfizer vaccines, 15 patients completed the vaccine series but were diagnosed less than 14 days after vaccination, and 54 patients were fully vaccinated [13]. In addition, population data from 14 US states on adults 65 years or older showed that among 7,280 COVID-associated hospitalizations, 75% of the cases were unvaccinated, 12% were partially vaccinated, and 5% were fully vaccinated [14]. Patients who were vaccinated and hospitalized with COVID-19 infection were more likely to be immune-compromised or have hypertension, diabetes, coronary artery disease, or chronic kidney disease [15].

A limited number of studies have described the characteristics of fully vaccinated patients with vaccine breakthrough infection. Of 371 patients admitted with COVID-19 infection, 82 out of 132 (62.1%) fully vaccinated individuals met the criteria for severe or critical COVID-19 infection [16]. Among hospitalized patients receiving two doses of the Pfizer-BioNTech vaccine, 66% required oxygen, and 13% required mechanical ventilation, with a mortality rate of 22% [17]. In a review of outcomes of 91 patients with breakthrough infections, 75 were partially vaccinated, and 16 were fully vaccinated. Of 91 vaccinated patients hospitalized with COVID-19, 14% required ICU admission [18]. Four of the 75 partially vaccinated and four of the 16 fully vaccinated patients died or were discharged to hospice [18]. There is still a lack of data comparing the clinical severity of COVID-19 infection and its outcomes among vaccinated individuals to those who were partially vaccinated or unvaccinated.

This study aimed to evaluate how the clinical features, risk factors, disease severity, and outcomes varied by vaccination status among individuals with COVID-19 infections requiring hospitalization.

## Materials and methods

### Study design

This investigation was a multicenter historical cohort study done by retrospective chart review. We reviewed the electronic medical records of hospitalized adult patients with a laboratory-confirmed diagnosis of COVID-19 from December 15, 2020, to October 29, 2022, at five Ascension Hospitals in southeast Michigan. Cases were identified using COVID-19-related ICD-10 codes and from the infection control database. ICD-10 codes included any of the following: U07.1, U07.2, J12.89, J20.8, J40, J22, J98.8 and J80 in conjunction with B97.29. Vaccination status was obtained through the Michigan Care Improvement Registry (MCIR) on the patient’s electronic medical chart. The Ascension Institutional Review Board approved the study.

### Data collection

Data collection included demographic data (age, race, and sex), body mass index (BMI), comorbidities, COVID-19 vaccination history, treatments given, in-hospital mortality, and length of hospitalization. Comorbid conditions were used to calculate the Charlson Comorbidity index [19]. We reviewed the medical records for patients’ clinical symptoms and signs, medications, laboratory findings on admission, and radiological assessments at hospital admission. Outcome measures, including length of hospital stay, intensive care unit (ICU) admission, length of ICU stay, and discharge disposition were also collected.

### Definitions

A confirmed case of COVID-19 was defined as a positive test result from a real-time reverse-transcriptase-polymerase-chain-reaction (RT-PCR) assay of nasopharyngeal swab specimens. “Fully vaccinated” described individuals two weeks after receiving two doses of monovalent Moderna or Pfizer-BioNTech vaccines or one dose of the Johnson and Johnson vaccines. “Partially vaccinated” described individuals two weeks after receiving one dose of the monovalent Moderna or Pfizer-BioNTech vaccine or less than 14 days after receiving the second dose of the monovalent Moderna or Pfizer-BioNTech vaccine or a single dose of the Johnson and Johnson vaccine [15]. “Unvaccinated individuals” had not received any vaccines against COVID-19 or less than 14 days after receiving the first dose of monovalent Moderna or Pfizer-BioNTech. “Breakthrough infection” describes the diagnosis of COVID-19 infection more than two weeks after being fully vaccinated. The degree of severity of COVID-19 (mild, moderate, severe, and critical) was determined using the WHO COVID-19 severity scale [20]. Patients with mild disease were symptomatic without evidence of viral pneumonia or hypoxia; patients with the moderate disease showed clinical signs of viral pneumonia but had oxygen saturation measured by a pulse (SpO_2_) ≥ 90% on room air; patients with the severe disease showed clinical signs for viral pneumonia plus either respiratory rate > 30 breaths per minute or had oxygen saturation measured by pulse oximetry (SpO_2_) < 90% on room air; patients with critical disease met criteria’s for acute respiratory distress syndrome (ARDS) or had acute life-threatening organ dysfunction with either the presence of altered mental status (delirium), evidence of pulmonary embolism, laboratory evidence of coagulopathy, or a requirement for mechanical ventilation.

COVID-19 pneumonia was defined as an acute respiratory disorder meeting at least three out of four criteria: respiratory signs/symptoms (cough/ dyspnea/ tachypnea), fever, oxygen saturation below 94%, and abnormal chest x-ray at hospital admission. Fever was defined as an axillary temperature of 37.5 °C or higher. Renal impairment was defined as mild if serum creatinine was 1.5-2-fold higher than baseline or severe if it was 2-fold higher than baseline or if the patient received hemodialysis or peritoneal dialysis. Patients with home steroid use were those receiving a steroid dose equivalent to at least 15 mg of prednisone per day for at least seven days consecutively before the time of hospitalization.

### Statistical analysis

Descriptive statistics were calculated to characterize the study group. Continuous variables were described with the mean and standard deviation or median with interquartile range (IQR). Categorical variables were described as frequency distributions. Univariable analysis was done using Student’s t-test to compare two independent means with normal distributions and analysis of variance followed by multiple pairwise comparisons using the Bonferroni correction of the p-value for the comparison of three or more independent means with normal distributions. (In any data tables, the results of the Bonferroni-corrected comparisons are shown only when the overall F test had a p-value less than 0.05). When continuous data were non-normally distributed, comparing two distributions was done using the Mann-Whitney U test and the comparison of three or more distributions using the Kruskal-Wallis test. The chi-squared test of independence was used to assess the association between two categorical variables. Univariable analysis was done using both the four-category WHO severity scale (mild, moderate, severe, critical) and a two-category scale composed of mild/moderate and severe/critical. The results from the four-category and two-category univariable analyses were similar. Multivariable analysis was done using binary logistic regression. For the multivariable analysis, the results from the univariable analysis of the two-category scale were used (data not shown). All data were analyzed using SPSS v. 29.0, and a p-value less than 0.05 was considered to indicate statistical significance.

## Results

We included 341 patients admitted with COVID-19 infection, with a mean age of 57.9 ± 18.3 years, 54.8% (187/341) female, and 48.7% (166/341) black/African American. Of these patients, 65.7% (224/341) were unvaccinated, 8.5% (29/341) had received one dose,22% (75/341) had received two doses, and 3.8% (13/341) had received three doses. The mean BMI of the cohort was 31.7 ± 10.1, and the median Charlson weighted index of comorbidity (CWIC) score was 0.0 (IQR 0.0, 2.0). The mean length of stay among the cohort was 6.7 ± 5.7 days. Mild disease was present in 29% of patients (99/341), 26.4% (90/341) had moderate disease, 33.4% (114/341) had severe, and 11.1% (38/341) had critical COVID infection based on the WHO categorization at the time of hospitalization. Forty-two patients (12.3%) required mechanical ventilation, 46 patients (13.5%) died during admission, and 22 (6.5%) required readmission to one of the study hospitals within 30 days of their initial discharge.

When comparing the characteristics of patients in the three vaccination groups (Table 1), unvaccinated patients were younger than fully vaccinated (p < 0.001) patients and were more likely to be black/African American (p = 0.002). While unvaccinated and partially vaccinated patients were more likely to have private insurance, fully vaccinated patients were more likely to have public insurance (Medicare) (p = 0.04). Partially vaccinated patients had significantly higher mean BMI levels (p < 0.001) than fully vaccinated and unvaccinated individuals. Obesity was present in 53% (181/341) of patients, and there was no significant difference in the distribution of obesity between the three vaccination groups. Fully vaccinated patients were more likely to have heart failure (18.2%) (p = 0.01), hypertension (72.7%) (p = 0.01), renal disease (22.7%) (p < 0.001), and malignancy (14.8%) (p = 0.05) than partially vaccinated and unvaccinated patients. There was no statistically significant difference in the other comorbidities among the three groups.Over one-third of partially vaccinated patients were self-reported current smokers (37.9%) compared to 12.5% of unvaccinated patients and 18.2% of fully vaccinated patients (p = 0.002).

**Table 1:** Characteristics of fully vaccinated, partially vaccinated, and unvaccinated hospitalized COVID-19 patients

Characteristic	Vaccination Status			p-value
	A. Unvaccinated (n = 224)	B. Partially Vaccinated (n = 29)	C. Fully Vaccinated (n = 88)	
Mean age (years)	54.8 ± 18.6	58.7 ± 15.9	65.4 ± 15.9	A vs. C* <0.001
**Sex**				0.11
Male Female	41.1% (92) 58.9% (132)	51.7% (15) 48.3% (14)	53.4% (47) 46.6% (41)	
**Race**				0.002
White Black/African American Other	39.7% (89) 52.2% (117) 8% (18)	37.9% (11) 58.6% (17) 3.4% (1)	62.5% (55) 36.4% (32) 1.1% (1)	
**Insurance Type**				0.041
Commercial Medicare Medicaid	129 (57.6%) 81 (36.2%) 14 (6.3%)	15 (51.7%) 12 (41.4%) 2 (6.9%)	35 (39.8%) 49 (55.7%) 4 (4.5%)	
Mean body mass index	31.1 ± 9.3	38.9 ± 17.1	30.8 ± 7.8	B vs. A; B vs. C. < 0.001
Obesity (n = 181)	114 (50.9%)	18 (62.1%)	49 (55.7%)	0.4
Median CWIC	0.0 (0.0, 1.75)	1.0 (0.0, 1.0)	1.0 (0.0, 3.0)	< 0.001
Prior MI (n = 43)	25 (11.2%)	2 (6.9%)	16 (18.2%)	0.2
CHF (n = 34)	16 (7.1)	2 (6.9%)	16 (18.2%)	0.01
PVD (n = 56)	32 (14.3)	5 (17.2%)	19 (21.6%)	0.3
CVD (n = 76)	44 (19.6)	6 (20.7)	26 (29.5)	0.2
Diabetes without complications (n = 76)	46 (20.5%)	10 (34.5%)	20 (22.7%)	0.2
Diabetes with complications (n = 30)	17 (7.6%)	2 (6.9%)	11 (12.5%)	0.4
Asthma (n = 28)	18 (8%)	4 (13.8%)	6 (6.8%)	0.4
COPD (n = 47)	31 (13.8%)	5 (17.2%)	11 (12.5%)	0.8
Renal disease (n = 40)	19 (8.5%)	1 (3.4%)	20 (22.7%)	< 0.001
Dementia (n = 21)	12 (5.4%)	1 (3.4%)	8 (9.1%)	0.4
Any malignancy (n = 29)	14 (6.3%)	2 (6.9%)	13 (14.8%)	0.05
HIV (n = 6)	3 (1.3%)	1 (3.4%)	2 (2.3%)	0.7
Hypertension (n = 203)	124 (55.4%)	15 (51.7%)	64 (72.7%)	0.013
Steroid Use at home (n = 10)	70% (7)	0% (0)	30% (3)	-
Current Smoker (n = 55)	28 (12.5%)	11 (37.9%)	16 (18.2%)	0.002
Drug Use (n = 13)	9 (4%)	1 (3.4%)	3 (3.4%)	0.9
**Vital signs on admission**				
Mean Temperature	37.2 ± 1.0	37.2 ± 0.9	37 ± 0.7	0.3
Mean Heart rate	101.1 ± 23.8	100.4 ± 21.9	91.2 ± 19.1	A vs. C 0.002
Mean Respiratory rate	20.3 ± 5.7	20.5 ± 5.3	19.6 ± 4.8	0.6
Mean Oxygen saturation	94.3 ± 5.9	95 ± 4.5	95.7 ± 5.3	0.1
**Laboratory findings on admission**				
Mean White blood cell count	7.7 ± 4.6	7.7 ± 5.1	8.7 ± 6.3	0.3
Mean Hemoglobin	12.8 ± 2.3	12.8 ± 2.9	12.1 ± 2.1	A vs. C 0.05
Mean Platelet count	228.7 ± 93.4	217.5 ± 81.8	219.9 ± 84.1	0.7
Mean Total protein	7.1 ± 1.1	6.7 ± 1	6.7 ± 0.9	A vs. C 0.01
Mean Albumin	3.6 ± 0.6	3.7 ± 0.6	3.6 ± 0.6	0.9
Mean C-reactive protein	90.1 ± 81	77.2 ± 68.6	65.4 ± 75.8	0.09
**Chest imaging on admission No. (%)**				
Abnormal chest x-ray (n = 198)	143 (71.1%)	16 (64%)	39 (48.8%)	0.002
Abnormal CT scan (n = 118)	89 (92.7%)	7 (77.8%)	22 (71%)	0.006

*Abbreviations*: n: Number, CWIC: Charlson weighted index of comorbidity, MI: Myocardial infarction, CHF: Congestive heart failure, PVD: Peripheral vascular disease, CVD: Cerebrovascular disease, COPD: Chronic obstructive pulmonary disease, HIV: Human immunodeficiency virus

^*^From multiple pairwise comparisons, Bonferroni correction of the p-value

^†^Kruskal-Wallis test

Table 1 describes the clinical features observed upon admission. Fully vaccinated patients had lower heart rates on admission (p = 0.002) compared to unvaccinated patients. Other vital signs, including temperature, respiratory rate, oxygen saturation, and blood pressure, were similar in the three groups. There were no significant differences in oxygen requirements at admission by vaccination group; however, at 24 h, the need for O_2_ supplementation was 60.3% in the unvaccinated group, 55.2% in the partially vaccinated group, and 43.2% in the fully vaccinated group (p = 0.024). Fully vaccinated patients had lower mean hemoglobin (p = 0.05) and total protein levels (p = 0.01) compared to unvaccinated patients. No differences between the three groups were observed in white blood cell counts, platelet counts, and C-reactive protein (CRP) levels. Creatinine phosphokinase (CPK), troponin, ferritin, and d-dimer levels were excluded from the analysis because many patients did not have these laboratory tests done.

Chest x-rays were done at the time of hospitalization in 88.8% (199/224) unvaccinated patients, 86.2% (25/29) partially vaccinated patients, and 90.9% (80/88) fully vaccinated patients. Of the 199 unvaccinated patients who had chest x-rays done, 71.1% had abnormal chest x-rays on admission compared to 48.8% in fully vaccinated and 64% in partially vaccinated patients (p = 0.002) (Table 1). The most common chest x-ray finding was patchy bilateral infiltrates present in 44.4% (135/304) of all patients who had x-rays done. A total of 135 patients had computed tomography (CT) imaging of the chest done on admission, of which 71.1% (96/135) were unvaccinated, 6.7% (9/135) were partially vaccinated, and 22.2% (30/135) were fully vaccinated (p = 0.2). Among unvaccinated patients who had a CT scan done, 92.7% had abnormal results compared to 71% in the fully vaccinated and 77.8% in the partially vaccinated patients (p = 0.006). The most common chest CT findings across all groups were ground glass opacities, 34.6% (47/135), and patchy bilateral infiltrates, 30.1% (41/135). Forty out of 47 patients with ground glass opacities and 32 out of 41 patients with patchy bilateral infiltrates were in the unvaccinated group.

**Fig. 1:**
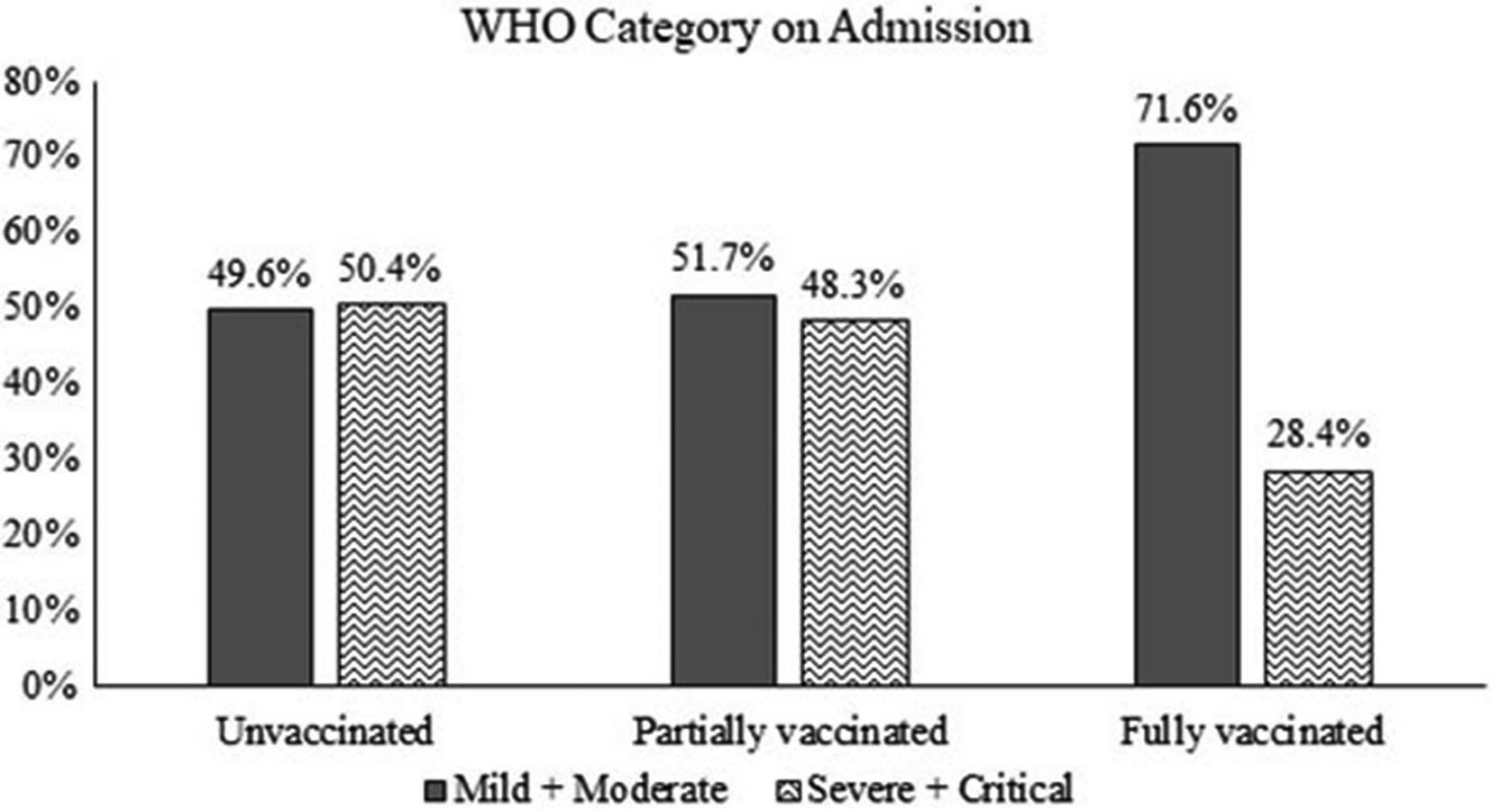
Disease severity among the fully vaccinated, partially vaccinated, and unvaccinated hospitalized COVID-19 patients on admission based on WHO classification

Severe and critical disease on admission based on the WHO classification (Fig. 1.) was observed in 50.4% of unvaccinated patients compared to 48.3% of partially vaccinated patients, and 28.4% of fully vaccinated patients (p = 0.002). Overall, fully vaccinated individuals had fewer complications and required fewer interventions during their hospital stay than unvaccinated and partially vaccinated individuals (Table 2). Partially vaccinated patients were more likely to require mechanical ventilation (p = 0.003) and ICU admission (p = 0.01) than unvaccinated and fully vaccinated individuals. Unvaccinated patients were more likely to develop septic shock (p = 0.05) and ARDS (p = 0.03) compared to partially and fully vaccinated patients. Most unvaccinated patients required steroids during their hospital stay compared to partially vaccinated patients and fully vaccinated patients (p < 0.001). Partially vaccinated patients were more likely to receive Remdesivir than unvaccinated and fully vaccinated patients (p < 0.001). Unvaccinated patients were more likely to receive IL-6 inhibitors (p = 0.04) than partially and fully vaccinated patients. Higher readmission rates were observed in fully vaccinated patients compared to unvaccinated and partially vaccinated patients; however, this difference was not statistically significant (p = 0.4). The case fatality rate in fully vaccinated patients was significantly lower than in unvaccinated and partially vaccinated patients.

**Table 2:** Treatment and outcomes among the fully vaccinated, partially vaccinated, and unvaccinated hospitalized COVID-19 patients

Complication	No. (%)			p value
	Unvaccinated (n = 224)	Partially vaccinated (n = 29)	Fully vaccinated (n = 88)	
Intubation/Mechanical ventilation (n = 42)	34 (15.2%)	6 (20.7%)	2 (2.3%)	0.003
Intensive Care Unit admission (n = 47)	38 (17%)	5 (17.2%)	4 (4.5%)	0.01
Septic shock (n = 29)	24 (10.7%)	3 (10.3%)	2 (2.3%)	0.05
Acute Respiratory Distress Syndrome (n = 23)	21 (9.4%)	1 (3.4%)	1 (1.1%)	0.03
Acute Kidney Injury (n = 84)	61 (27.2%)	7 (24.1%)	16 (18.2%)	0.2
Renal Replacement Therapy (n = 8)	5 (2.2%)	1 (3.4%)	2 (2.3%)	0.9
Any Arrhythmia (n = 30)	22 (9.8%)	2 (6.9%)	6 (6.8%)	0.7
Atrial arrythmia (n = 26)	18 (8.0%)	2 (6.9%)	6 (6.8%)	0.9
Steroids given (n = 222)	162 (72.3%)	19 (65.5%)	41 (46.6%)	< 0.001
Remdesivir given (n = 108)	81 (36.2%)	13 (44.8%)	14 (15.9%)	< 0.001
Interleukin-6 Inhibitor given (n = 30)	25 (11.2%)	3 (10.3%)	2 (2.3%)	0.04
Readmission within 30 days (n = 22)	12 (6.5%)	1 (3.8%)	9 (10.6%)	0.4
Death (n = 46)	40 (17.9%)	3 (10.3%)	3 (3.4%)	0.003

When assessing risk factors associated with the severity of COVID-19 disease (Table 3), patients with severe disease were significantly older than those with mild disease (p = 0.004), and a patient with severe disease tended to have a higher BMI than those with mild disease (p = 0.06). Fully vaccinated patients and patients of black/African American race were significantly more likely to have mild or moderate disease at the time of hospitalization. Fully vaccinated black/ African American patients were less likely to have severe disease compared to fully vaccinated white patients (15.6% vs. 36.4%, p = 0.04). In addition, higher respiratory rate on admission, higher mean WBC, and higher absolute neutrophil count were associated with severe and critical infection compared to mild infection. There was a significant association between median creatinine on admission and disease severity (p = 0.004). The presence of hypertension (0.06) and home steroid use (0.09) were higher among patients with more severe categories of disease compared to those with mild infection at the time of hospitalization. Serum albumin levels showed an inverse association with the severity of COVID-19 at the time of hospitalization, i.e., lower albumin levels were associated with severe and critical disease.

**Table 3:** Univariable analysis of risk factors among hospitalized COVID-19 patients using WHO severity scale at the time of admission

Characteristic	A. Mild	B. Moderate	C. Severe	D. Critical	p value
	n = 99	n = 90	n = 114	n = 38	
Mean Age (yrs.)	52.5 ± 22.1	58.4 ± 17.9	61.0 ± 15.4	61.3 ± 13.8	A vs. C, p = 0.004
Mean BMI	29.3 ± 7.5	32.0 ± 11.1	32.9 ± 10.1	33.4 ± 12.4	A vs. C, p = 0.06
Median CWIC score	0.0 (0.0, 2.0)	1.0 (0.0, 2.0)	0.0 (0.0, 2.0)	0.0 (0.0, 2.0)	0.32
Mean Respiratory Rate on Admission	18.6 ± 3.5	19.3 ± 4.1	21.0 ± 5.7	23.6 ± 8.7	A vs. C, p = 0.006; A vs. D, p < 0.001; B vs. D, p < 0.001, C vs. D, p = 0.06
Mean WBC Count	7.6 ± 3.6	8.0 ± 6.7	7.5 ± 4.1	10.4 ± 6.2	A vs. D, p = 0.025; C vs. D, p = 0.012
Mean Absolute Neutrophil Count	5.4 ± 3.3	5.3 ± 3.8	5.9 ± 3.8	8.4 ± 5.9	A vs. D and B vs. D, p < 0.001, C vs. D p = 0.006
Median Creatinine on Admission	0.9 (0.8, 1.2)	1.0 (0.8, 1.4)	0.9 (0.7,1.4)	1.3 (1.0, 1.7)	0.004
**Race**					< 0.001
White	34.3% (34)	45.6% (41)	56.1% (64)	42.1% (16)	
Black	61.6% (61)	51.1% (46)	38.6% (44)	39.5% (15)	
Other	4.0% (4)	3.3% (3)	5.3% (6)	18.4% (7)	
Smoking	17.2% (17)	23.3% (21)	13.2% (15)	5.3% (2)	0.12
Hypertension	48.5% (48)	64.4% (58)	64.9% (74)	60.5% (23)	0.06
Steroid Use (Home)	1.0% (1)	1.1% (1)	6.1% (7)	2.6% (1)	0.09
Mean Serum Albumin	3.9 ± 0.73	3.6 ± 0.52	3.5 ± 0.49	3.4 ± 0.60	A vs. B, p = 0.012; A vs. C & A vs. D, p < 0.001
**Vaccination group**					0.005
None	53.5% (53)	64.4% (58)	71.1% (81)	84.2% (32)	
Partial	10.1% (10)	5.6% (5)	8.8% (10)	10.5% (4)	
Complete	36.4% (36)	30.0% (27)	20.2% (23)	5.3% (2)	

*Abbreviations* n: Number, BMI: Body mass index, CWIC: Charlson weighted index of comorbidity, WBC: White blood cells

As stated in the methods section, results from the univariable analysis using the two-category scale were used to determine which variables to enter the multivariable logistic regression model. Variables that were included in defining the WHO severity categories were excluded as independent variables. The initial variables that went into the model included age at admission, BMI, heart rate on admission, albumin value, initial absolute lymphocyte count on admission, vaccination group, race, elevated creatinine from baseline, hypertension, and home steroid use. Using a forward stepwise likelihood ratio algorithm, the final model included age at admission, BMI, albumin, vaccination group, race, and home steroid use (Table 4).

**Table 4:** Multivariable analysis of risk factors for breakthrough severe COVID-19 infections requiring hospitalization among the fully vaccinated, partially vaccinated, and unvaccinated individuals

Characteristic	Odds Ratio	p-value	95% CI
Age at admission	1.02	0.02	1.004, 1.04
Fully vaccinated^*^	0.19	<0.001	0.09, 0.38
Black/African American race^**^	0.39	0.001	0.22, 0.70
Albumin	0.51	0.007	0.31, 0.83
BMI	1.04	0.005	1.01, 1.08
Steroids use (Home)	7.5	0.03	1.3, 44.9

*Abbreviations* CI: Confidence interval, BMI: Body mass index

^*^Unvaccinated is the comparator category

^**^White race is the comparator category

From the model, after controlling for age, BMI, albumin, race, and home steroid use, fully vaccinated patients were 5.3 times (p < 0.001) less likely to have severe/critical disease. Notably, after controlling for vaccination status and the other variables in the model, Black patients were 2.6 times less likely to have severe/critical disease (p = 0.001), and every unit increase in albumin reduced the risk of severe/critical disease by 96% (p = 0.007). Older patients (p = 0.02), patients with higher BMI values (p = 0.005), and those who were using steroids for seven consecutive days at the time of hospitalization (p = 0.03) were more likely to have severe/critical disease after controlling for the other variables in the model.

## Discussion

Our study showed that fully vaccinated patients had more mild disease, fewer complications during their hospital stay, and a lower-case fatality rate compared to partially vaccinated and unvaccinated patients. The majority of our patients (247 vs. 69) were in the time frame that corresponded to the circulation of the Omicron variant (December 2021- October 2022) compared to the Delta variant (June-November 2021. In addition, we identified six factors independently associated with severe and critical COVID-19 infection based on WHO classification; these factors were vaccination status, age, race, BMI, home steroid use and serum albumin levels on admission.

Patients who were fully vaccinated had more comorbidities, which are known risk factors for breakthrough infections and severe disease in general. Our cohort of fully vaccinated patients were more likely to be white and had a higher mean age. They also were more likely to have hypertension, heart failure, renal disease, and malignancy compared to unvaccinated and partially vaccinated patients. This study is similar to the one conducted in Israel on 152 vaccinated COVID-19 hospitalized patients, but some comorbid conditions were less frequent in our study [17]. These included heart failure (18.2% vs. 28%), kidney disease (22.7% vs. 32%), and cancer (14.8% vs. 24%). In contrast to the previously mentioned study, diabetes and lung disease were not associated with being fully vaccinated [17]. Vaccinated patients also had lower mean hemoglobin and total protein levels, suggesting increased comorbid conditions predisposing them to more severe infection and requiring hospital admission. In vaccinated patients, comorbidities are associated with reduced immunological responses to infection or immunization, thereby putting them at risk for breakthrough infections [21]. This risk can be curtailed by administration of additional booster vaccine doses.

Severe COVID-19 infection based on the WHO classification was lowest in the fully vaccinated group, followed by partially vaccinated, then the unvaccinated group, suggesting a protective effect of mRNA vaccines against severe disease. Higher readmission rates in fully vaccinated patients may be explained by the fact that fully vaccinated patients were more likely to be older and have comorbidities. Most of the unvaccinated patients had abnormal chest imaging on admission, were more likely to require mechanical ventilation and use of steroid therapy, and experienced higher rates of death compared to the fully vaccinated group. These findings are consistent with the study by Tenforde et al., which found that patients who died or required mechanical ventilation were less likely to be vaccinated [22]. Of note, partially vaccinated patients were more likely to require ICU admission and mechanical ventilation compared to unvaccinated and fully vaccinated patients. Many of the risk factors associated with severe COVID-19 among unvaccinated individuals in our study were similar to those reported in the literature, including advanced age, higher BMI, hypertension, and creatinine on admission [23–26].

In our study, low serum albumin levels on admission were associated with higher risks for severe/ critical disease. Every unit increase in albumin level reduced the risk of severe/ critical disease by 96%. Albumin, WBC, absolute lymphocyte and neutrophil counts, AST, and creatinine are the serum biomarkers associated with severe disease in unvaccinated patients [25, 27]. Albumin is a negative acute phase reactant synthesized in the liver. It is associated with severe COVID-19 infection and poor outcomes [25]. Studies hypothesize that the mechanism of hypoalbuminemia is not linked to liver injury as it was not associated with changes in AST and ALT [25, 28, 29]. In contrast, results of a systematic review published in 2021 showed higher AST and ALT levels to be independently associated with poor outcomes [30]. Our study showed an association between having severe and critical COVID-19 infection and serum AST levels but not ALT [25]. C-reactive protein, CPK, D dimer, and LDH were reported by multiple studies to be associated with the severity of illness in patients with COVID-19 [31, 32]. Our study did not show a difference in the CRP, CPK, D dimer, or LDH levels when comparing patients in the two severity categories.

Our study showed that black/African American patients were 2.4 times less likely to be in the severe category than whites (OR = 0.04, p = 0.002). This is in contrast with the current evidence that shows that black/African American people had a higher risk of hospitalization, need for mechanical ventilation, and death [33]. Hospitalized black/African American patients in our study were younger than whites and, thus, might have less severe disease. Our study also finds that patients who were using steroid doses equivalent to at least 15 mg of prednisone per day for at least seven days consecutively at the time of hospitalization were more likely to have severe/ critical disease. Higher odds of hospitalization had been reported with the use of glucocorticoid therapy at prednisone-equivalent doses ≥ 10 mg/ day among COVID-19 patients with rheumatic disease [34]. Systemic steroids can blunt the initial innate immune response with the potential of augmenting viral replication in the early phase of the disease, putting patients atrisk for hospitalization and severe COVID-19 infection [35]. Severe clinical disease manifesting at the time of hospitalization among individuals on steroid therapy could be from opportunistic infections, coinfections, or life-threatening side effects, likely hyperglycemia [36, 37].

## Limitations

Our study is retrospective; some study variables, such as laboratory biomarkers, were only done on a subset of patients, preventing accurate data analysis. We did not have enough data available to conduct an analysis based on the type of vaccine and the variant of concern. Also, no information was available about the prior treatments and prophylaxis that admitted patients might have received.

## Conclusion

Patients who were unvaccinated or partially vaccinated had more in-hospital complications, severe disease, and death as compared to fully vaccinated patients. Factors associated with severity include advanced age, obesity, low serum albumin, and steroid use.

## Data Availability

The data set was gathered from the study site and is not accessible to the public.
